# Sensitivity of PS/CoPd Janus particles to an external magnetic field†

**DOI:** 10.1039/d1ra02410h

**Published:** 2021-05-10

**Authors:** Anna Eichler-Volf, Yara Alsaadawi, Fernando Vazquez Luna, Qaiser Ali Khan, Simon Stierle, Chi Xu, Michael Heigl, Zahra Fekri, Shengqiang Zhou, Peter Zahn, Manfred Albrecht, Martin Steinhart, Artur Erbe

**Affiliations:** Helmholtz-Zentrum Dresden-Rossendorf, Institute of Ion Beam Physics and Materials Research Bautzner Landstrasse 400 Dresden Germany a.eichler-volf@hzdr.de; Institute of Chemistry of New Materials, Osnabrueck University Barbarastr. 7 Osnabrueck Germany; Institute of Physics, University of Augsburg Universitaetsstrasse 1 Augsburg Germany

## Abstract

The dual nature of Janus particles confers fascinating properties such as a response to multiple stimuli. In this communication, we systematically study the sensitivity to a uniform external magnetic field of isolated Janus rod-shaped and spherical particles in water confined to two dimensions. The Janus asymmetry of the particles is given by magnetic [Co(0.28 nm)/Pd(0.90 nm)]_8_ multilayer films deposited onto monodisperse polystyrene (PS) nanorods and microspheres, respectively. It is shown that the particles dispersed in water respond to weak magnetic field applied in in-plane direction. Here we demonstrate that a precise control of the in-plane particle orientation can be obtained for magnetic field strengths higher than 0.1 mT for microspheres and 0.4 mT for nanorods.

## Abstract 2

PS/CoPd Janus particles respond very sensitively to application of low external magnetic fields. Owing to the magnetic properties, the PS/CoPd particles may be used, for example, to sense the presence of weak magnetic fields as micro-magnetometers.

## Graphical abstract

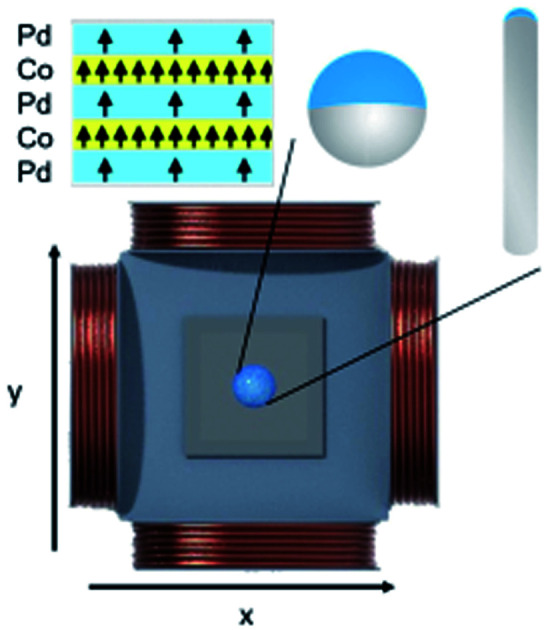

## 1 Introduction

The very sensitive response of soft matter to external stimuli, like temperature, pH, electric and magnetic fields, causes strong research interest. The small-scale motion control has attracted wide-spread attention in various fields including chemistry, material science, biology and medicine.^1–4^ Exposing colloids to external fields, for example, electric,^5^ magnetic,^6^ optical^7^ and acoustic^8^ ones, gives the fascinating opportunity to study soft matter in a controlled way. Magnetostatic interaction is widely independent from temperature fluctuations, surface charges and the pH-value of the environment. Moreover, the magnetic field can penetrate into non-magnetic or weakly magnetic materials that are used for fabricating microfluidic devices or biological matter. Therefore, micro- and nano-objects in closed environments can be effectively manipulated without being directly contacted. Within the past decade, researchers have developed a variety of techniques for synthesizing colloidal magnetic particles with a wide range of reproducible shapes and sizes that differ in the distribution of the magnetic material within each particle. Magnetic nanoparticles with isotropic magnetization distribution have been fabricated *via* chemical synthesis,^9–11^ electrodeposition^12^ and lithographic techniques.^13^ Single particles with distinct magnetic properties can be used as basic building blocks for complex structures and applications. Magnetic nanoparticles have also been embedded in hydrogels or polymers *via* microfluidic channels or by encapsulation.^14–16^ Narrow size distribution of the magnetic nanoparticles is important, since the blocking temperature of nanomagnets depends critically on the size of the particles. A polydisperse sample results in broad blocking temperature unwanted in applications. Another synthesis method relies on the surface modification of polymer or silica particles *via* metal deposition. Janus particles, named after the two-faced Roman god Janus, are characterized by surface areas with different polarities or functionalities. By using this approach, one part of the particle surface is covered with a composition containing Co,^17,18^ Ni^19^ or Fe^20,21^ metals providing ferromagnetic properties. Magnetic properties of Janus particles depend strongly on their size and shape in addition to their intrinsic magnetic characteristics, such as magnetic moment and anisotropy. The dynamics of a magnetic active Janus particle undergoing Brownian motion under the influence of a uniform magnetic field has been analytically and numerically examined by several groups.^22,23^ The interaction of a magnetic field with the magnetic dipole of a particle induces a torque that competes with the randomization of its orientation due to rotational diffusion. Most of the experimental studies dealing with magnetic control of spherical active Brownian particles have been focused on monitoring their spatial trajectory. In this work, we focus on the monitoring of the particle orientation in dependency of the magnetic field strength. To our knowledge, there is no systematic experimental study on the sensitivity of the orientation of magnetic Janus particles to low external magnetic fields.

Here we introduce spherical and rodlike magnetic Janus particles. The Janus particles are based on polystyrene (PS) microspheres with a 4.2 μm in diameter or nanorods with a diameter of 0.4 μm and a length of 5 μm. The hemispherical ferromagnetic coating by Co/Pd multilayers possesses a perpendicular magnetic anisotropy. For the spherical Janus particles, one half of their surface was covered by magnetic material, while for Janus nanorods, the metal film was deposited onto one tip only and covered 4% of the total rod surface. The sensitivity of artificially designed magnetic particles and clusters dispersed in water has been studied *via* video microscopy. The magnetic-field sensitivity is defined by the fluctuations of the particle orientation in low magnetic fields. The reorientation time of Janus particles was studied as a function of magnetic field strength. Both types of particles demonstrated a high sensitivity to low magnetic fields. The PS/CoPd microspheres follow exactly the direction of the magnetic field starting from 0.1 mT. A reasonable response of the nanorods was observed for fields starting from 0.4 mT.

## 2 Materials and methods

### 2.1 Synthesis of PS/CoPd Janus particles

#### 2.1.1 Preparation of nanorods with hemispherical tip shape

**Fig. 1:**
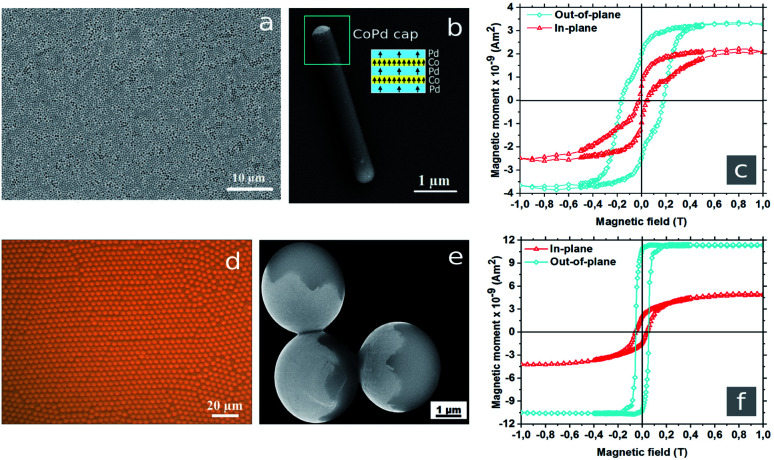
(a) SEM image of the AAO template with embedded PS nanorods aligned parallel to each other with one side opened to the air. (b) Single Janus PS/CoPd nanorod with a diameter of 400 nm and a length of 5 μm after metal deposition followed by removing the AAO template. The structure of magnetic caps consisting of Co/Pd multilayers as inset is schematically shown in blue and yellow colors. The arrows indicate the local magnetic moment in the layers. (c) Hysteresis loops obtained for Co/Pd capped nanorods in horizontaly (in-plane) and vertically (out-of-plane) applied magnetic fields. (d) Optical micrograph of monodisperse PS microspheres with diameter of 4.2 μm after evaporation of the solvent forming densely packed two-dimensional arrays. (e) SEM image of PS/CoPd Janus microspheres with hemispherical metallic caps (brighter areas). (f) Hysteresis loops measured with a SQUID magnetometer for Janus microspheres along in-plane and out-of-plane directions.

The PS nanorods with hemispherical tips were fabricated in two steps following the procedure reported earlier.^24^ Briefly, as a first step, a 1 : 10 solution of PS (*M*_w_ = 24 700 g mol^−1^*M*_w_/*M*_n_ = 1.03, Polymer Source Inc., Canada) in toluene was infiltrated into the nanopores of a homemade anodic aluminum oxide (AAO) membrane template^25^ with a pore diameter of 0.4 μm and a pore depth of 5 μm. To complete the infiltration, AAO membranes with PS bulk film were annealed at 160 °C for 6 hours under Ar while applying a load of 0.3 bar. Then, the samples were cooled to room temperature at a rate of −5 °C min^−1^ for homogeneity and thermal relaxation of the PS film. In the second step, the separation of nanorods from the PS film with the subsequent formation of the hemispherical tips, was performed by modification of procedures reported earlier.^26,27^ The samples were immersed in a polyethylene glycol (PEG) 400 bath at 80 °C for 1 h to induce dewetting of PS. To complete dewetting process, the samples were annealed at 200 °C for approximately 4 h in a PEG 400 bath. Finally, the AAO templates with infiltrated PS nanorods (Fig. 1a) were washed in an ethanol/water mixture, at least 5 times followed by drying at 40 °C for 18 h.

#### 2.1.2 Fabrication of PS microsphere arrays

The Janus spherical particles were prepared according to a previous study.^28^ A 2.5 wt% aqueous solution of PS microspheres with diameter of 4.2 μm was purchased from Bangs Laboratories, Inc. and used as received. A monolayer of PS microspheres was prepared by drop-casting method using pre-cleaned n-doped Si-wafers with areas of 2 cm^2^ (Fig. 1d). Then, the Co/Pd multilayers were deposited on the top of microsphere arrays.

#### 2.1.3 Metal deposition

A magnetic thin film was sputtered normal to the samples represented by either nanorod arrays or monolayers of PS microspheres *via* magnetron sputter deposition at a base pressure of 2 × 10^−8^ mbar and the Ar gas pressure of 3.5 × 10^−3^ mbar at room temperature. The deposition rates were kept at 0.03 nm s^−1^ for Ta, 0.05 nm s^−1^ for Pd and 0.03 nm s^−1^ for Co, respectively. As a first step, to improve the adhesion and the perpendicular magnetic anisotropy of thin metal films, the samples were covered by a seed-layer consisting of Ta(3.0 nm)/Pd(3.0 nm).^29^ Then, a magnetic multilayer sequence consisting of [Co(0.28 nm)/Pd(0.9 nm)]_8_ was deposited. As a final step, the film was capped with an additional Pd(2.1 nm) layer to prevent oxidation. The resulting deposited composition was Ta(3 nm)/Pd(3 nm)/[Co(0.28 nm)/Pd(0.9 nm)]_8_/Pd(2.1 nm). The resulting film thickness was 17.54 nm. Finally, the magnetically capped particles were saturated at 1 T for 5 min at room temperature to align the cap moments in the out-of-plane direction.

#### 2.1.4 Compositional characterization

The morphological and compositional characterization of the Janus particles were performed by Scanning Electron Microscopy (ZEISS Auriga, Germany) equipped with an Energy Dispersive X-ray Spectrometer (Oxford Instruments, UK). The samples were prepared by dropping 2 μL Janus particle solutions onto glass substrates previously cleaned with oxygen plasma. The measurements were performed with in-lens and X-ray detectors, acceleration voltage of 14 kV and 5 mm working distance. Prior to the imaging tests, the samples were sputter coated with Pt/Ir at 20 mA for 15 s three times in a K575X Emitech device.

#### 2.1.5 X-ray diffraction measurements

The X-ray diffraction was performed on a diffractometer system XPERT-PRO (PANalytical, the Netherlands) having sample stage MRD cradle configuration. The Cu Kα radiation source, *λ* = 0.15418 nm was used. The measurement was conducted from start 2*Θ* angle of 10° till end angle of 90° values with a step size of 0.02. The total time for measurements were about approximately 9 hours.

### 2.2 Sample preparation

#### 2.2.1 Releasing of nanorods from AAO template

The AAO template was etched in a 5 wt% aqueous KOH solution. After releasing, the nanorods were washed with deionized water (DI) under slow steering with a shaker at a speed of 30–40 rounds per minute (rpm). Nanorods were washed, at least, 8 times with DI water until the pH of suspension reached values less than 7.

#### 2.2.2 Detaching of microspheres

The microspheres were separated from the Si/SiO_2_ wafer in ultrasonic water bath within 20–30 min at room temperature. Then, the particles were collected and resuspended in DI water.

#### 2.2.3 Sample cell

Few droplets of the suspended Janus particles were placed on a pre-cleaned glass slide and left for particles sedimentation. Once particles were settled down, the external magnetic field was applied.

### 2.3 Video recording and analysis

The trajectories and orientations of particles were studied by a Leica DMI3000B transmission microscope integrated with a Leica DFC395 CCD video camera and a 63X objective with a numerical apperture (NA) of 0.70. Two pairs of Helmholtz coils with diameter of 2 cm^2^ provided homogeneous magnetic fields in *x* and *y* directions. All videos were captured at a frame rate of 20 frames per second (fps). The particle movements were subsequently analyzed by homemade Python and LabView scripts. The video processing algorithm was used to track the positions and the orientations of individual nanorods and microspheres with pixel resolution. 1 pixel corresponds to a size of 0.127 μm^2^.

### 2.4 *ζ*-potential measurements

The *ζ*-potential measurements were performed using a Malvern Zetasizer Nano ZSP instrument equipped with a 632 nm HeNe laser operating at a 173° detector angle. Prior to the experiment, the particles were dispersed in water. Three and five tests were performed for each sampleset, respectively.

## 3 Results and discussion

The geometric shape of the PS/CoPd particles changes negligibly after the deposition of 17.54 nm thick magnetic film (Fig. 1b and e). The hemispherical metallic caps follow the curvature of the particles. Due to directed deposition, the film thickness varies across the cap following a cosine behavior with the maximum at the center of the cap. It was shown that below a critical Co layer thickness of about 0.8 nm, the magnetization points perpendicular to the film plane due to interface anisotropy.^30^ Transition of easy axis from perpendicular (out-of-plane) to a parallel (in-plane) direction occurs at a Co thickness of about 0.8 nm.^31^ For a Co film thickness of 0.28 nm as deposited here, a planar film exhibits perpendicular anisotropy. The total magnetic moment depends weakly on the thickness of the Pd layers in the multilayer.^32^ Magnetic force microscopy (MFM) measurements at a plane of Co/Pd multilayer deposited on Si confirmed the perpendicular magnetic anisotropy. The magnetization orientation also follows the curvature of the particle. The magnetization decays from the top to the rim of the cap and hence there is a preferred magnetic direction – along the symmetry axis of the cap.

In order to find the magnetic saturation point, 10 μL suspension were placed onto Si-wafer with area of about 10 mm^2^. After the evaporation of water, the sample was measured at temperatures of 5 K and 300 K, respectively. The SQUID measurements at a temperature of 300 K are represented in Fig. 1c and f. For the out-plane orientation of the external magnetic field, the saturation for nanorods was achieved at 0.5 T. In turn, the Janus microspheres reached magnetic saturation at 0.2 T. For Janus particles easy axis of magnetization was found in out-of-plane.

**Fig. 2:**
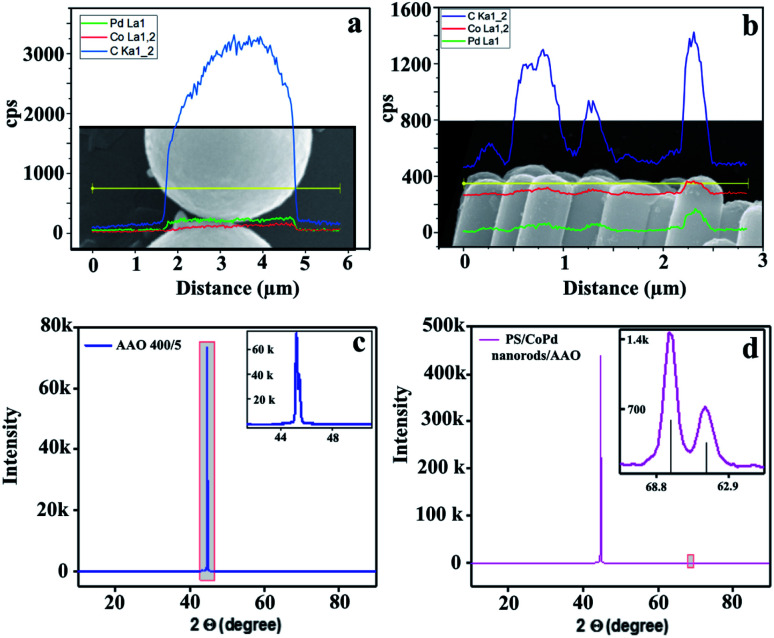
(a) Elemental composition derived from spatially resolved EDX spectra of PS/CoPd microspheres and (b) nanorods. (c) XRD measurements for empty AAO reference and (d) nanorods with metal multilayer thin film.

To identify the elemental composition of Janus particles, a spatially resolved EDX characterization was performed. The EDX spectra directly revealed the presence of the atomic elements in the sample as shown in Fig. 2a and b. The system was left to automatically detect the elements and the following elements were determined in this way Si, O and C. Manually we have checked if one of the following is also present: Pd and Co. Pd and Co were registered in small amounts. Additionally, we performed X-ray diffraction to analyze the structure of the metal thin film. The Janus nanorods embedded in AAO membrane with the capped side facing to the air were used for analysis. Empty AAO template with a pore diameter of 400 nm and a pore depth of 5 μm was used as a reference. The XRD pattern of empty alumina is presented in Fig. 2c. The diffraction peak position was recorded as the detector angle, 2*Θ*. The XRD pattern shows the hexagonal structure of AAO with a preferential orientation along the (200) direction. For PS/CoPd nanorod arrays embedded in AAO template, two peaks were identified as shown in Fig. 2d. Here, the peak at 44.6° corresponds to (200) plane reflection of AAO template.^33^ The double peak at 69° corresponds to (220) planes reflection of CoPd alloy.^34,35^

As the next step, the particles were suspended in deionized water. The *ζ*-potential measurements on the rodlike particles showed −35.1 ± 7.3 mV (5 measurements), while the average value of *ζ*-potential on the microspheres was −45.6 ± 2.7 mV (3 measurements). Hence, the Janus particles in contact with the aqueous solution were negatively charged. To encapsulate the particle dispersion for long time from the environment, a droplet of about 3–5 μL of particle suspension was enclosed between two glass slides separated by a distance of several ten micrometers. Then, the glass slides were sealed with UV glue to prevent evaporation of the solvent. Due to the density mismatch and the electrostatic repulsion, the particles settled within a few nanometers above the bottom glass surface. When the Janus nanorods were sedimented in water, the Co/Pd caps pointed in random directions, while the Co/Pd caps of microspheres were oriented downwards to the glass slide. Applying sufficient magnetic field, Co/Pd caps reoriented and aligned to the magnetic field.

**Fig. 3:**
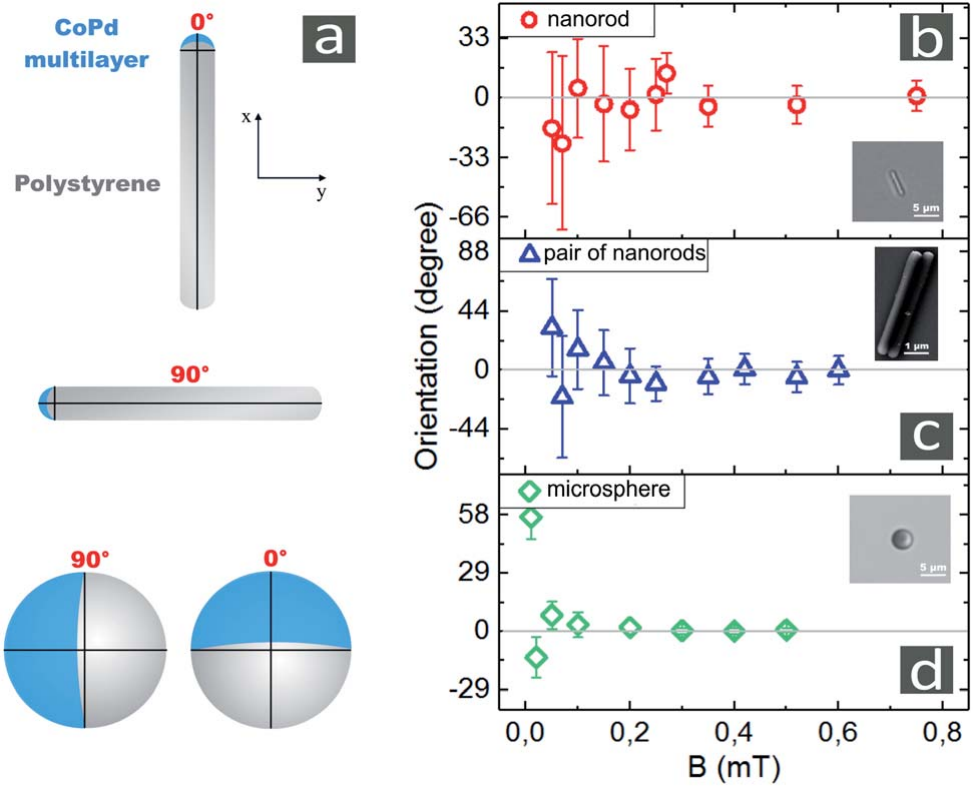
(a) Schematic representation of Janus particles with one side coated by a metallic cap. Sensitivities of single Janus nanorods (b), cluster consisting of two rods (c) and microspheres (d) to externally defined applied magnetic field. The data represent average measurements for 4–6 different single particles and 1 cluster. All observations were taken over 240 s.

Fig. 3a represents a schematic illustration of spherical and rodlike Janus particles with one magnetic cap. The orientation of the cap is defined by a polar angle *θ* formed by the magnetic field direction and the macrospin orientation. In other words, *θ* identifies the rotational displacement of Janus particle in a polar direction. Depending on the field strength, the orientation of the macrospin of the Co/Pd cap fluctuates around the direction of the external field *B*. For *B* applied along *y* axis, the fluctuations around the externally defined direction are shown in Fig. 3b–d.

We measured the orientation of Co/Pd caps for each value of the magnetic field strength *B*, at least, over 60 s. Prior to setting a different value of *B*, we applied *B*_*y*_ = 0 for, at least, 30 s to orient the caps randomly. As can be seen in Fig. 3b for single nanorods, the values of standard deviations decay with increasing *B*. For weak *B* in the range of 0.05–0.4 mT, the standard deviations varied from Δ*θ* = 48° to Δ*θ* = 20° around the externally defined orientation *θ* = 0. For *B* = 0.4 mT, the uncertainty of the cap position was about Δ*θ* = 10.5°. These results indicate that single nanorods align with fields starting from 0.4 mT. For *B* in the range of 0.4–0.8 mT, Δ*θ* slowly decayed. The variation of angles for *B* = 0.8 mT was obtained as Δ*θ* = 8.3°.

The van-der-Waals interaction between the parallel aligned nanorods leads to formation of the closed-packed clusters consisting of particles. The smallest cluster comprises two parallel nanorods as its constituents. The results of application of a magnetic field to the pairs of nanorods are shown in Fig. 3c by the mean value of *θ* and fluctuation Δ*θ*. In magnetic field of 0.1 mT, the fluctuation of *θ* was about 31°, and dropped down to about Δ*θ* = 10° at 0.8 mT. The Janus microspheres kept their orientation by static magnetic field of 0.1 mT nearly perfectly as depicted in Fig. 3d by the fluctuation Δ*θ* of 6.3°. With increasing magnetic field strength *B* = 0.4 mT, the fluctuations from a fixed orientation was quenched to approximately 1°.

**Fig. 4:**
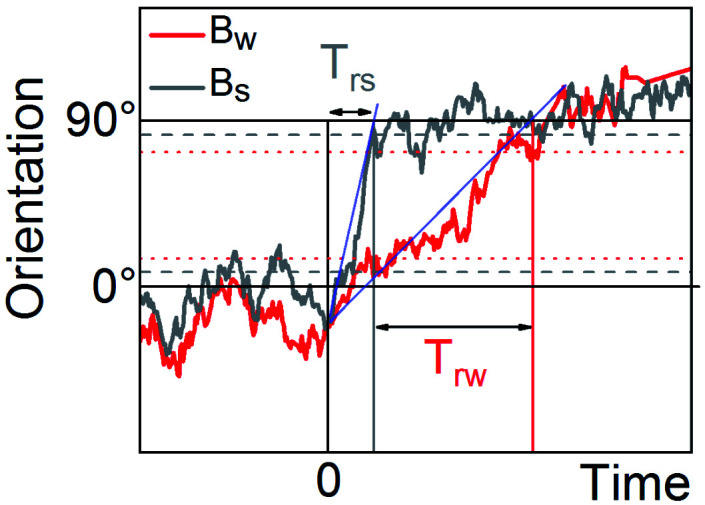
The 90-degree reorientation of Janus nanorods in weak *B*_w_ and strong *B*_s_ and magnetic fields. The experimental trajectories measured at 0.5 mT and 0.9 mT are shown in red and grey, correspondingly. Blue lines correspond the fits to the transition region of the trajectories. The dotted lines show the standard deviations for weak and strong magnetic fields.

**Fig. 5:**
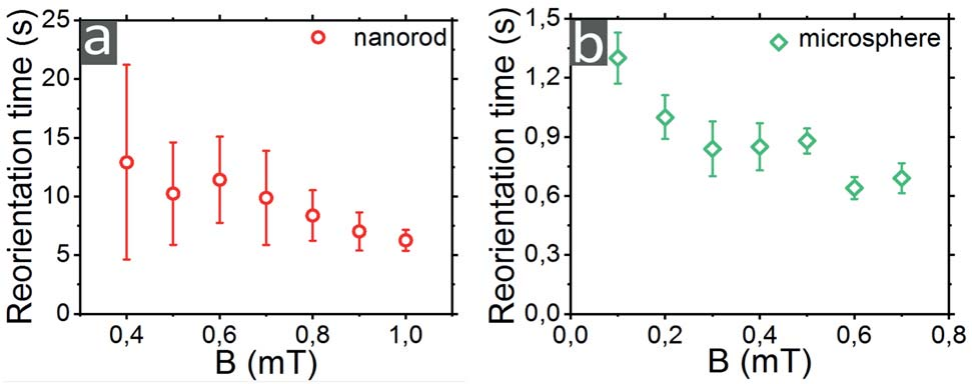
Reorientation times of (a) single Janus nanorods and (b) microspheres as a function of magnetic field. Each data point on the graph is an average result of 10 measurements.

The reorientation times *T* of particles in weak (*T*_rw_) and strong (*T*_rs_) magnetic fields are shown in Fig. 4. Here, the particle was rotated by 90° by switching the external magnetic field at constant amplitude from *x* to *y* direction. The reorientation time is defined between the crossing of the linear fit of the transition region of the trajectory with the 0° and 90° levels. The intervals *T*_rs_ and *T*_rw_ might have a different left border. If the orientation of particles for certain field reached values *B*_w_ ± Δ*B*_w_ or *B*_s_ ± Δ*B*_s_ and then remained in this range, at least, for 2 s, we considered the response of particle as a reorientation. The reorientation times for single Janus nanorods and microspheres are summarized in Fig. 5.

A 90-degrees reorientation time of particle decreased upon the increase of *B*. We found that the reorientation time of Janus nanorods changes from 12.9 ± 8.3 s for *B* = 0.4 mT to 6.2 ± 0.9 s for *B* = 1 mT, correspondingly. In the presence of strong magnetic field of *B* = 1 mT, the reorientation of rodlike particle occurred about two times faster than that for *B* = 0.4 mT. The reorientation time of PS/CoPd spherical particles was one order of magnitude smaller than that measured for nanorods. The microspheres were rotated by 90° within 1.3 ± 0.13 s in a weak magnetic field of *B* = 0.1 mT, while in a strong field of *B* = 0.7 mT the reorientation was observed in 0.69 ± 0.08 s.

### 3.1 Brownian motion in a presence of magnetic field

Brownian motion is the result of random collisions between a microscopic particle and the molecules of the surrounding fluid. The presence of external constant magnetic fields influences the random, uncontrolled movement of ferromagnetic Janus particles in a fluid due to the interplay between Brownian diffusion and magnetic torques. In turn, the magnetic field causes a confining potential of the angular motion and the Brownian particle is captured in the local minimum (lowest energy state) of a potential well. In the most simplest case, the Brownian particle behaves like a one-dimensional stochastically damped harmonic oscillator.^36,37^ The width of the potential energy function depends on the strength of the magnetic field and the magnetic moment of the cap. Moreover, the width of the potential function decreases with the increase in the magnetic field strength. Classically, the Brownian motion at temperature *T* is restricted to the region where the confinement potential is less than *k*_B_*T*, with *k*_B_ the Boltzmann constant. At a constant temperature, if the magnetic field is weak, the particle moves in a broad region while for strong fields the region becomes more narrow. Therefore, the orientation of magnetic particle directed parallel to strong field *B*_s_ deviates less from the field direction than that for weak field *B*_w_.

**Fig. 6:**
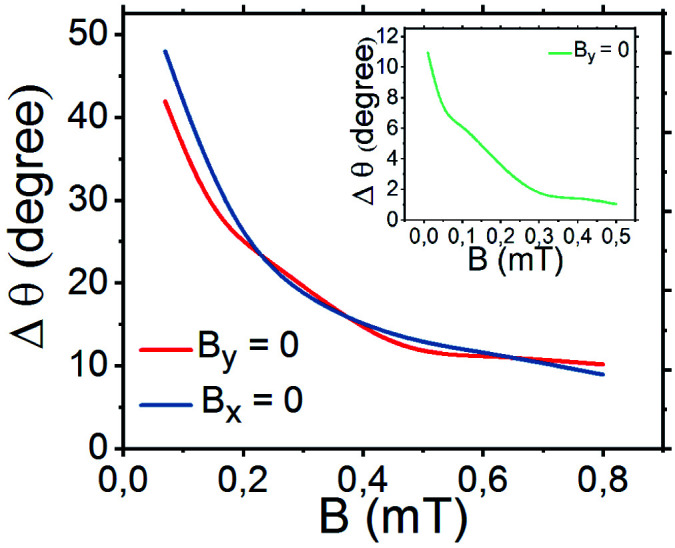
The standard deviation of polar angle *θ* for single Janus nanorods and microspheres (shown as an inclusion).

Fig. 6 shows the field-dependent standard deviation functions, when the magnetic field points vertically (*B* along *y* direction) or horizontally (*B* along *x* direction). These results have been obtained for Janus rod-like particles. As can be seen from the graph, the two functions have nearly the same profile indicating the equivalence of the responses of Janus nanorods to vertically or horizontally applied magnetic fields. As expected, Δ*θ* decreases with the field strength. For a weak magnetic field, the Brownian diffusion dominates over magnetic interaction. For a strong magnetic field, the thermal fluctuations of the orientational motion of the particle was nearly suppressed, and the magnetic dipole was aligned along the field direction. Due to the larger magnetic moment, the alignment is much stronger for the microspheres with respect to the nanorods.

### 3.2 The influence of anisotropy of PS/CoPd Janus particles on the rotational dynamics

The cap significantly impacts the rotational dynamics of the particle due to gravitational torque and the electrostatic torque resulting from the asymmetry in electrostatic forces. The gravitational torque on a Janus particles is caused by the density mismatch between the cap and native particle. The gravitational torque depends on the weight of the cap, which in turn depends on the thickness, total size and cap material. In our experiments, the thickness of Co/Pd caps was fixed at 17.54 nm for both types of Janus particles. The estimation of weight of non-coated PS spherical and rodlike particles gives 40.9 × 10^−15^ kg and 0.64 × 10^−15^ kg, respectively. The spherical particle without coating is approximately 64 times heavier than nanorods. The mass of the CoPd caps amounts to approximately 2.8 × 10^−15^ kg for microspheres and 0.026 × 10^−15^ kg for nanorods, respectively. Hence, the mass of the cap amounts to about 7% for the spheres and about 4% for the rods. After the deposition of the Co/Pd multilayer, the caps of spherical particles in an aqueous solution were mostly oriented downwards similar to Rashidi *et al.*^38^ The authors investigated the impact of particle size and coating thickness on the magnitude of rotation for Au-coated PS spherical Janus particles with radii ranging from 1 to 6 μm and a cap thicknesses up to 20 nm. It was shown that the magnitude of downward rotation decreased with increasing particle diameter at a fixed cap thickness. We estimated the hydrodynamic sizes (*d*_h_) of Janus nanorods and microspheres. *d*_h_ of spherical particles equals to 4.2 μm while *d*_h_ for nanorods is 1.8 μm. The calculation of *d*_h_ for nanorods was based on the equivalent sphere theory^39^1
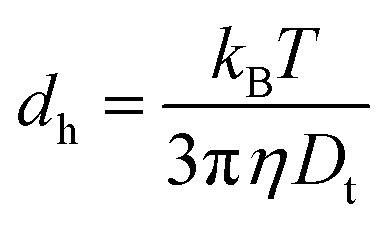
where *T* is temperature, *η* is the viscosity of water. The translational diffusion coefficient for rodlike particles *D*_t_ = 0.24 (μm)^2^ s^−1^ was estimated based on the Tirado and Garcia de la Torre theory.^40^ In line with the Brownian dynamics simulation of Rashidi *et al.*, in our experiments, we also observed that the amplitude of fluctuation of *θ* for spherical particles was much smaller than that for nanorods at fixed magnetic fields.

### 3.3 The impact of Co/Pd cap size on the sensitivity to magnetic fields

To investigate the influence of cap size on the sensitivity, we calculated the ratio between the Co/Pd cap surfaces and the PS parts for spherical and rodlike particles based on the SEM images. For Janus microspheres, the ratio is estimated to be 0.5 (half of particle is coated with Co/Pd). For nanorods, only one of the hemispherical tips with radius of 0.2 μm was coated with Co/Pd. The total surface of the nanorod can be represented by the side of the cylinder, which when unrolled is a rectangle with sides of 5 μm and 1.256 μm, and the surface of the sphere with radius of 0.2 μm. The ratio of the metalized surface of the rodlike particle to the total surface area constitutes was determined as small as 0.04. The Co/Pd surface area of the cluster pair increased by a factor two as compared to a single particle. At the same time, the polymeric surface area is 1.7 times higher compared to a single nanorod. The ratio between metal-coated and nonreactive surface areas is approximately 0.05, 20% higher than that of a single particle. Despite of the tiny amount of magnetic materials, Janus nanorods were observed to be highly sensitive to application of weak magnetic fields. Co/Pd magnetic capped particles revealed that Janus microspheres were more sensitive to magnetic fields as compared to single nanorods. The reason might be the larger amount of ferromagnetic material on the microspheres, and the larger size of the nanorods with respect to the magnetically active caps.

## 4 Conclusion

We synthesized CoPd-capped PS Janus particles, which respond very sensitively to application of low external magnetic fields. The results reveal that a uniform magnetic field affects the dynamic behavior of magnetic active particles. The precise control of the orientation of the particles was achieved at 0.1 mT for microspheres and at 0.4 mT for nanorods, respectively. The rotation time of 90 degrees of Janus nanorods decreases by a factor of 2 upon increasing the magnetic field strength from 12.9 ± 8.3 s at 0.4 mT to 6.2 ± 0.9 s at 1 mT. The reorientation time values of microspheres were one order of magnitude smaller than that for nanorods and amounted to 1.3 ± 0.13 s at 0.1 mT and 0.69 ± 0.08 s at 0.7 mT. Owing to the magnetic properties, the PS/CoPd particles may be used, for example, to sense the presence of weak magnetic fields as so-called micro-magnetometers.

## Author contributions

A. E.-V. and A. E. conceived and planned the experiments. A. E.-V., F. V. L., Y. A., M. H., Q. A. K., Z. F., C. X. and S. S. carried out the experiments. A. E.-V. and Y. A. analysed the data. A. E.-V. wrote the manuscript with support from A. E., M. S., S. Z., M. A. and P. Z. All authors have reviewed the manuscript.

## Conflicts of interest

There are no conflicts to declare.

## Supplementary Material

RA-011-D1RA02410H-s001

RA-011-D1RA02410H-s002

RA-011-D1RA02410H-s003

RA-011-D1RA02410H-s004

RA-011-D1RA02410H-s005

RA-011-D1RA02410H-s006
